# Isolation, Characterization, and Quantification of Bacteria from African Sausages Sold in Nairobi County, Kenya

**DOI:** 10.1155/2018/3861265

**Published:** 2018-10-23

**Authors:** W. H. Karoki, D. N. Karanja, L. C. Bebora, L. W. Njagi

**Affiliations:** Department of Veterinary Pathology, Microbiology and Parasitology, University of Nairobi, P.O. Box 29053-00625, Nairobi, Kenya

## Abstract

African sausages are local popular delicacies in Kenya. Demand for these sausages has resulted in this delicacy's vendors being on the increase. However, health risk posed to unsuspecting consumers of African sausages sold in informal, unhygienic make shift road-side kiosks in major cities of Kenya is largely unknown. A descriptive study was designed to isolate, characterize and quantify bacteria from African sausages sold in Nairobi County. A total of hundred (100) African sausages (62 roasted and 38 nonroasted) were conveniently collected from three meat eatery points of Westlands, Kangemi slum, and Pangani estates. Five genera of bacteria, namely,* Staphylococcus* spp. at 50.4%,* Bacillus* spp. at 19.5%,* Streptococcus *spp. 9.8%,* Proteus *spp. 2.4%, and* E. coli* spp. at 1.6%, were isolated from 80 African sausage samples. The total aerobic bacterial count range was between 1.0-9.9x10^1^ and 1.0-9.9x10^7^ log cfu/g with 37 samples having total aerobic bacterial count of between 1.0-9.9 x10^4^ and 1.0-9.9 x10^7^ log cfu/g. There was no significant difference (p>0.05) in distribution of isolates and total aerobic bacterial count across geographical sites studied among the roasted and nonroasted African sausages. This study has demonstrated presence of bacteria in African sausages which are potentially zoonotic to humans. Comprehensive study is needed to sample more eatery meat points in Nairobi and other areas in order to demonstrate pathogenic attributes of these isolates and establish the respective total aerobic bacterial count. There is also need to establish the sources of bacteria due to high total aerobic bacterial count determined in the current study.

## 1. Introduction

African sausages, popularly known as Kenyan sausages locally known as “Mutura” in Kikuyu dialect, is a local delicacy for low, middle income earners and beer drinkers. It is a protein rich meat snack comprising goat or cow cleaned intestines stuffed with cooked small pieces of meat and formed into long coils; sometimes blood is added. Processed African sausages are then placed in boiling water or soup for 30 to 40 minutes and then roasted over coals on outdoor grills using low to medium heat and turning frequently to dehydrate the meat and give it the sensational smoky taste. The internal temperature should be at least 160 degrees Fahrenheit [1].

Animal proteins such as meat, meat products, and even blood are regarded as high-risk perishable commodities with respect to pathogen content, natural toxins, and other possible contaminants [2]. Among the bacteria isolated from animal products in recent studies include* Staphylococcus aureus*,* Streptococcus* sp.,* Escherichia coli*,* Campylobacter jejuni*,* Clostridium perfringens*,* Shigella* sp., and* Salmonella* sp.

Consumption of food with such microbial pathogens and toxins is estimated to result in approximately 1.5 billion episodes of diarrhea and over 3 million deaths globally each year [3]. Increase in demand for finger-licking African sausages has resulted in this delicacy's vendors being on the increase, especially in slum areas [4]. These informal businesses mostly operate in unhygienic makeshift and road-side meat points of Nairobi and major cities in Kenya, where they are unregulated and the standardized preparation of these African sausages is disregarded.

In regard to Torok* et al*. [5] study, food-borne disease from African sausages arises from intestinal bacteria or external contamination, which is a result of unhygienic food preparation especially if the vendor fails to adhere to hygiene practices during processing, preparation, handling, and/or storage; it may therefore pose serious health risk to unsuspecting consumers resulting in outbreaks of food-borne illnesses. Results of this study will help the relevant regulatory body in laying down food safety measures for the African sausages.

## 2. Materials and Methods

### 2.1. Study Design

A descriptive study design was employed whereby a convenience sampling of retail meat outlets from Westlands, Pangani, and Kangemi was carried out. One hundred (100) African sausages samples (38 nonroasted and 62 roasted) were collected, processed, isolated, characterized, and quantified.

### 2.2. Study Area

The study was carried out in Nairobi County which is one of the 47 counties of Kenya [6]. It is the smallest yet most populous county, the capital and largest city of Kenya, which has experienced one of the most rapid growths in urban centres with a population of 3,375,000 as at year 2009 census [7]. It has a total area of 696 km^2^ with 17 parliamentary constituencies. Nairobi is a cosmopolitan and a multicultural city.

Economically, it can be subdivided into three main categories: (1) the high end or leafy suburbs or upper-class estates, the likes of Muthaiga, Karen, Westlands, among others, (2) the Middle-class estates of Pangani, Buruburu, among others, and (3) low class estates of Mukuru, Mathare, Kangemi slums, among others [8]. Three ready-to-eat vending sites and meat eatery points of Westlands market, Kangemi market, and Pangani estate were conveniently selected on the basis of easy access, perceived sanitation, and relative hygiene levels. The number of vendors in these areas is not known; but they tend to converge around the shopping areas.

### 2.3. Sample Size Calculation

Prevalence of common meat contaminants in previous studies was used to determine the sample size required to detect the presence of the bacteria. An expected prevalence rate of between 7% was used to estimate the sample size in this study since similar studies [9–12] reported a prevalence rate of between 3 and 14%. Using the above information, the sample size was calculated using the formula: n=z^2^pq/d^2^ where n is the desired sample size, Z is the standard normal deviate set at 1.96, p is the estimated prevalence, q=1-p, and d is the degree of accuracy set at 0.05 as given by Fisher et al. [13].

### 2.4. Homogenate Preparation

At the laboratory, one-gram portions of the African sausages (roasted and nonroasted) were obtained aseptically from the vendors, picked separately as they are sold using sterile glass bottles, stored on ice before processing in the laboratory within 24 hours of collection, cut into small pieces on a sterile chopping board using a sterile knife, and blended (homogenized) in 4ml of 0.1% peptone water to obtain 1:5 initial dilution.

### 2.5. Bacterial Isolation and Characterization

Since the researcher suspected presence of coliforms and other fastidious organisms, the homogenates of the African sausages were streaked on general purpose enriched medium (blood agar) and selective and differential medium for members of family Enterobacteriaceae (MacConkey agar) (Oxoid Ltd. Thermo Scientific, UK) and incubated aerobically at 37°C for 24 hours. The isolated bacteria were identified based on colony morphology, Gram staining reaction, and biochemical characteristics using established standardized methods according to Bergey's Manual of determinative bacteriology [14].

### 2.6. Quantification of Total Aerobic Bacterial Count of the African Sausages

For the determination of bacterial load (total bacterial count), method given by Miles and Misra [15] was used. Serial dilutions of 10^−1^ to 10^−10^ were prepared from the African sausage homogenate stock solution that was prepared earlier. Nutrient agar plate was divided into four quadrants, and each quadrant served as one plate. Using a 25 *μ*l calibrated dropper (equivalent to 1/40^th^ of an ml), one drop from each dilution tube was placed per quadrant; each dilution was done in quadruplicate.

The drop was then allowed to dry and the plate incubated aerobically at 37°C for 24 hours [16], after which the number of colonies that grew per drop was counted using Quebec Dark Field colony counter taking the average count for the quadruplicate drops of each dilution. The concentration of the original bacterial suspension was then calculated and expressed as colony forming units per millilitre (cfu/ml), using the formula, a x 40 x 10^y^, where a is the average number of colonies in the 4 drops of one dilution tube/diluted suspension, 40 is the number of drops that make one millilitre (the drop being equivalent to 1.40^th^ of a ml), and 10^y^ is the dilution factor of the respective dilution tube/diluted suspension. This is then multiplied by 5, the initial dilution at homogenization stage.

### 2.7. Statistical Analysis

The experiment was done in triplicate. The means for the prevalence of bacterial isolates across the three geographical sites and the bacterial counts were compared by a Paired-Samples T Test. Differences were considered statistically significant when P<0.05. Statistical analysis was conducted using the software SPSS 13.0 (SPSS, Chicago, Illinois, USA)

## 3. Results

### 3.1. Isolation and Characterization

**Figure 1:**
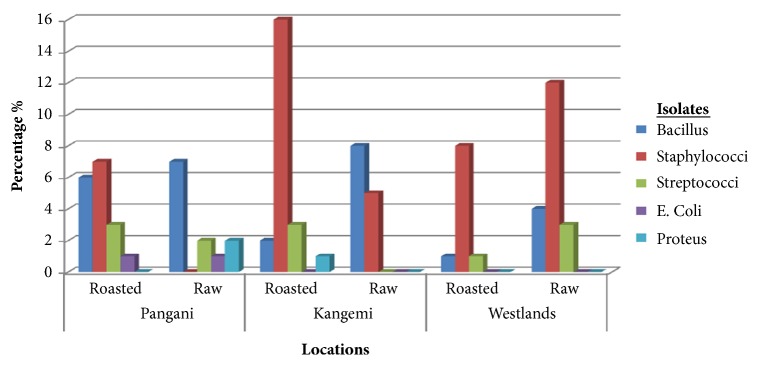
Prevalence of the five genera of bacteria isolated from African sausages sampled from Pangani, Kangemi, and Westlands estates, Nairobi County, Kenya: n=100.

Figure 1 gives results on the prevalence of isolated and characterized bacterial isolates. Five genera of bacteria (123 isolates) were isolated and characterized from 80/100 (80%) roasted and nonroasted African sausages. They were* Staphylococcus, Bacillus *spp.,* Streptococcus* spp.,* Proteus *spp., and* Escherichia coli. Staphylococci *spp. were the most predominant bacteria in all the sausage samples collected with a prevalence of 50.4% (62/123),* Bacillus* spp. at 19.5% (24/123),* Streptococcus *spp. 9.8% (12/123), and* Proteus *spp. 2.4% (3/123) while* E. coli *was isolated at 1.6% (2/123). With respect to roasted African sausages,* Staphylococcus* spp. accounted for 53.6% (15/28) of the isolates in Kangemi, 52.2% (12/23) in Pangani, and 38.1% (8/21) in Westlands.* Bacillus *spp. organisms were isolated at 7.1% (2/28) in Kangemi, 26.1% (6/23) in Pangani, and 4.8% (1/21) in Westlands; Streptococcus 10.7% (3/28) in Kangemi, 13% (3/23) in Pangani, and 4.8% 3/21) in Westlands; Proteus 3.6% (1/28) in Kangemi and 0% both in Pangani and in Westlands;* E. coli*, 4.3% (1/23) in Pangani and 0% both in Kangemi and in Westlands.

With respect to nonroasted African sausages,* Staphylococcus* spp. accounted for 50% (6/12) of the isolates in Kangemi, 45.5% (10/22) in Pangani, and 64.7% (11/17) in Westlands.* Bacillus* spp. organisms were isolated at 41.7% (5/12) in Kangemi, 31.8% (7/22) in Pangani, and 17.6% (3/17) in Westlands;* Streptococcus* spp. 0% in Kangemi, 9.1% (2/22) in Pangani, and 17.6% (3/17) in Westlands;* Proteus* spp., 9.1% (2/22) in Pangani and 0% both in Kangemi and in Westlands areas; E*. coli*, 4.5% (1/22) in Pangani and 0% both in Kangemi and in Westlands areas.  Table 1 gives the mean distribution of bacterial isolates. There was no significant difference (p>0.05) in distribution of isolates across the geographical areas under study.

### 3.2. Total Aerobic Bacterial Count from Roasted African Sausage

**Figure 2:**
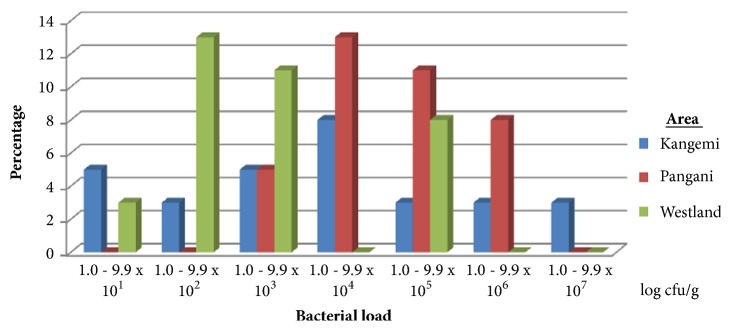
Percent total aerobic bacterial counts of the sampled roasted African sausages sold in the three study sites: n=100.

The results are as given in Figure 2. 22/62 (35.5%) roasted African sausage samples had a total aerobic bacterial count of between 1.0 and 9.9 x10^1^ log cfu/g, 11/62 (17.7%) samples had a total aerobic bacterial count of between 1.0 and 9.9 x10^2^ log cfu/g, 12/62 (19%) samples had a total aerobic bacterial count of between 1.0 and 9.9 x10^3^ log cfu/g, 9/62 (14.5%) samples had a total aerobic bacterial count of between 1.0 and 9.9 x10^4^ log cfu/g, 5/62 (8%) samples had a total aerobic bacterial count of between 1.0 and 9.9 x10^5^ log cfu/g, 2/62 (3%) samples had a total aerobic bacterial count of between 1.0 and 9.9 x10^6^ log cfu/g, and 1/62 (1.6%) sample had a total aerobic bacterial count of between 1.0 and 9.9 x10^7^ log cfu/g.

With respect to individual study sites, 10/24 (41.7%) of samples from Kangemi, 3/19 (15.8%) from Pangani, and 9/20 (45%) from Westlands area had a total aerobic bacterial count of between 1.0 and 9.9 x10^1^ log cfu/g. 1/24 (4.17%) of samples from Kangemi, 3/19 (15.8%) from Pangani, and 7/20 (35%) from Westlands area had a bacterial load of between 1.0 and 9.9 x10^2^ log cfu/g. 4/24 (16.7%) of samples from Kangemi, 7/19 (36.8%) from Pangani, and 1/20 (5%) from Westlands area had a bacterial load of between 1.0 and 9.9 x10^3 log^ cfu/g. 3/24 (12.5%) of samples from Kangemi, 4/19 (21%) from Pangani, and 2/20 (10%) from Westlands area had a bacterial load of between 1.0 and 9.9 x10^4^ log cfu/g. 3/24 (12.5%) of samples from Kangemi, 2/19 (10.5%) from Pangani, and 0% from Westlands area had a bacterial load of between 1.0 and 9.9 x10^5^ log cfu/g. 2/24 (8.3%) of samples from Kangemi, 0% from Pangani and Westlands area had a bacterial load of between 1.0 and 9.9 x10^6^ log cfu/g. 1/24 (4.17%) of samples from Kangemi, 0% from Pangani and Westlands area had a bacterial load of between 1.0 and 9.9 x10^7^ cfu/g.  Table 2 gives the mean total aerobic bacterial count from roasted African sausages across the three geographical areas. There was no significant difference (p≥0.05) in mean total aerobic bacterial count across the areas under study.

### 3.3. Total Aerobic Bacterial Count from Nonroasted African Sausages

**Figure 3:**
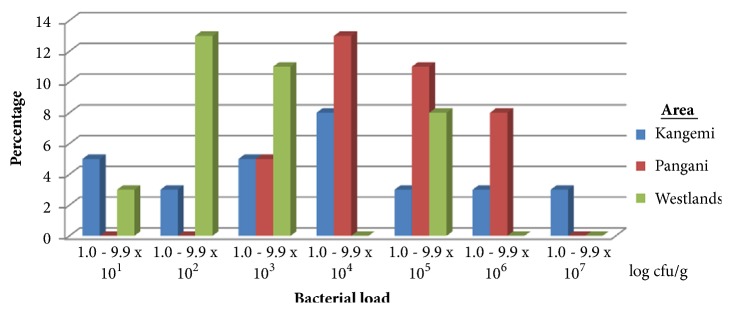
Percent total aerobic bacterial counts of the sampled nonroasted African sausages sold in the three study sites: n=100.

The results are as given in Figure 3. 3/38 (7.9%) nonroasted African sausage samples had a total aerobic bacterial count of between 1.0 and 9.9 x10^1^ log cfu/g, 6/38 (15.79%) samples had a total aerobic bacterial count of between 1.0 and 9.9 x10^2^ log cfu/g, 8/38 (21%) samples had a total aerobic bacterial count of between 1.0 and 9.9 x10^3^ log cfu/g, 8/38 (21%) samples had a total aerobic bacterial count of between 1.0 and 9.9 x10^4^ log cfu/g, 8/38 (21%) samples had a total aerobic bacterial count of between 1.0 and 9.9 x10^5^ log cfu/g, 4/38 (10.5%) samples had a total aerobic bacterial count of between 1.0 and 9.9 x10^6^ log cfu/g, and 1/38 (2.6%) sample had a total aerobic bacterial count of between 1.0 and 9.9 x10^7^ log cfu/g. With respect to individual study sites, 2/11 (18.8%) of samples from Kangemi, 0% from Pangani, and 1/13 (7.7%) from Westlands area had a total aerobic bacterial count of between 1.0 and 9.9 x10^1^ log cfu/g. 1/11 (9%) of samples from Kangemi, 0% from Pangani, and 5/13 (38.5%) from Westlands area had a total aerobic bacterial count of between 1.0 and 9.9 x10^2^ log cfu/g. 2/11 (18%) of samples from Kangemi, 2/14 (14.3%) from Pangani, and 4/13 (30.8%) from Westlands area had a total aerobic bacterial count of between 1.0 and 9.9 x10^3^ log cfu/g. 3/11 (27.3%) of samples from Kangemi, 5/14 (35.7%) from Pangani, and 0% from Westlands area had a total aerobic bacterial count of between 1.0 and 9.9x10^4^ log cfu/g. 1/11 (9%) of samples from Kangemi, 4/14 (28.6%) from Pangani, and 3/13 (23%) from Westlands area had a total aerobic bacterial count of between 1.0 and 9.9 x10^5^ log cfu/g. 1/11 (9%) of samples from Kangemi, 3/14 (21.4)% from Pangani, and 0% from Westlands area had a total aerobic bacterial count of between 1.0 and 9.9 x10^6^ log cfu/g. 1/11 (9%) of samples from Kangemi and 0% from Pangani and Westlands area had a total aerobic bacterial count of between 1.0 and 9.9 x10^7^ log cfu/g.  Table 3 gives the mean total aerobic bacterial count from nonroasted African sausages across the three geographical areas. There was no significant difference (p≥0.05) in mean total aerobic bacterial count across the areas under study.

## 4. Discussion

The data obtained on isolation and characterization of bacteria in the present study where* Staphylococcus* spp. and* Bacillus *spp. were the predominant isolates concurs on some aspects with reports by Oluwafemi and Simisaye [17] and Okonko* et al*. [18] working on beef sausages. However, differences are noted whereby in the present study* Streptococci *spp.,* Proteus *spp., and* E. coli* were isolated while in the latter,* Enterobacter, Pseudomonas, *and* Klebsiella* species were isolated from beef sausages and seafood, respectively.

The current study showed* Staphylococcus* species prevalence of 50.4%. This was lower than the Staphylococcus species recovery at 58.6% from hotels, restaurants, and cafes, report by Berynestad and Granums [19]. A study by Yusuf* et al*. [20], on percent occurrence of bacteria isolated from the “balangu” meat product in relation to all the retail outlets, reported a lower prevalence of* Staphylococcus aureus *at 12.5%. A study by Aycicek* et al*. [21] reported that processed foods were found to be more prone to* Staphylococcus* species contamination. This may have been attributed to contamination from aerial spores carried in the air, throat, hands, and nail of food handling persons [22].

The results of the roasted African sausages in current study contrast the findings by Orogu and Oshilim [22] who reported a higher prevalence (30%) of* Bacillus* from suya meat. However, similar prevalence was obtained by a study by Matos* et al*. [23] working with dry smoked sausages. Presence of* Bacillus *contamination in some of the samples examined in this study might have resulted from contamination from vendor's skin or the environment.

Similar finding on the prevalence of* Streptococcus* spp. was reported by Onuora* et al*. [24] working on grilled beef. The prevalence of* E. coli* reported in present study was slightly lower than that reported by Syne* et al*. [25] and Onuora* et al*. [24].* E. coli* presence in African sausages has the potential to cause diarrhea.

The incidence of* E. coli* obtained in this study is a cause for public health concern as this bacterium has been implicated in cases of gastroenteritis [26]. The presence of* Proteus* isolates was remarkably higher in a study by Gwinda* et al*. [27] from beef meat, compared to what was found in the current study. The presence of* Proteus *organisms in the meat samples can obviously be attributed to unhygienic food processing.

*Staphylococcus *spp.*, Bacillus *spp.,* Streptococcus *spp., and* E. coli* are known to produce potent enterotoxins and the ingestion of food containing these toxins can cause a sudden onset of illness within three to four hours, with nausea, vomiting, and diarrhea as the major symptoms [28]. There was no significant difference (p>0.05) in distribution of these organisms across the three geographical sites studied. In the present study, it was observed that there was no significant difference (p>0.05) in the total aerobic bacterial count across the three geographical sites studied. Total aerobic bacterial count of between 1.0-9.9 x 10^2^ and 1.0 x 9.9 10^4^ log cfu/g was reported in most 54/100 (54%) of African sausage samples.

Similar studies by Oluwafemi and Simisaye [17] reported a total aerobic bacterial count level of between 1.3 × 10^4^ and 4.0 × 10^8^ log cfu/g in beef sausage samples. In related studies, Inyang* et al*. [29] reported a total viable count of between 3.7 x 10^5^ to 2.4 x10^6^ log cfu/g while total viable count by Onuora* et al*. [24] reported a plate count of between 0.9 x 10^4^ log cfu/g and 1.5 x 10^4^ log cfu/g.

The difference in the total bacterial counts may be attributed to the samples used, unhygienic method of transportation, handling, processing, unhygienic environment, and practices such as dirty cutting boards and knifes or utensils. Cheesbrough [28] noted that insects also contribute to contamination by mechanical transfer of microorganisms to food products since they are left uncovered and exposed to dust.

There was no significant difference (p>0.05) in total aerobic bacterial count across the three geographical sites under study. This study therefore concludes that roasted and nonroasted African sausages sold in meat outlets in Nairobi County are contaminated with* Staphylococcus, Bacillus, Streptococcus, Proteus, *and* E. coli *organisms and poses food safety risks to the consumers. The presence of these organisms in ready-to-eat African sausages is a pointer that these African sausages were either processed under poor hygienic and sanitary conditions and insufficient processing or could have been from the animal intestines. Food safety enforcement authority therefore need to scale up inspection of establishments where African sausage vendors are.

## Figures and Tables

**Table 1:** Evaluation of the mean distribution of bacterial isolates from African Sausages across the three geographical areas using paired sample t-test (*P* values evaluated at 95% confidence limits).

		Paired Differences					t	df	Sig. (2-tailed)
		Mean	Std. Deviation	Std. Error Mean	95% Confidence Interval of the Difference				
					Lower	Upper			
Pair 1	Kangemi - Pangani	-1.333	4.590	1.874	-6.150	3.483	-.712	5	.509
Pair 2	Kangemi - Westlands	.333	2.160	.882	-1.934	2.600	.378	5	.721
Pair 3	Pangani - Westlands	1.667	6.282	2.565	-4.926	8.259	.650	5	.544

**Table 2:** Evaluation of the mean total aerobic bacterial count from roasted African sausages across the three geographical areas using paired sample t-test (*P* values evaluated at 95% confidence limits).

		Paired Differences					t	df	Sig. (2-tailed)
		Mean	Std. Deviation	Std. Error Mean	95% Confidence Interval of the Difference				
					Lower	Upper			
Pair 1	Kangemi - Pangani	147885.000	694148.169	159248.512	-186683.708	482453.708	.929	18	.365
Pair 2	Kangemi - Westlands	175895.500	658535.387	147252.989	-132308.548	484099.548	1.195	19	.247
Pair 3	Pangani - Westlands	26479.211	60777.503	13943.315	-2814.608	55773.029	1.899	18	.074

**Table 3:** Evaluation of the mean total aerobic bacterial count from nonroasted African sausages across the three geographical areas using paired sample t-test (*P* values evaluated at 95% confidence limits).

		Paired Differences					t	df	Sig. (2-tailed)
		Mean	Std. Deviation	Std. Error Mean	95% Confidence Interval of the Difference				
					Lower	Upper			
Pair 1	Kangemi - Pangani	-32490.000	1839501.213	554630.484	-1268283.730	1203303.730	-.059	10	.954
Pair 2	Kangemi - Westlands	504456.364	1407287.946	424313.281	-440972.543	1449885.270	1.189	10	.262
Pair 3	Pangani - Westlands	457091.538	851423.040	236142.264	-57418.255	971601.332	1.936	12	.077

## Data Availability

The data used to support the findings of this study are available from the corresponding author upon request.
